# Notch ankyrin domain: evolutionary rise of a thermodynamic sensor

**DOI:** 10.1186/s12964-022-00886-4

**Published:** 2022-05-18

**Authors:** Filip Vujovic, Neil Hunter, Ramin M. Farahani

**Affiliations:** 1 IDR/Westmead Institute for Medical Research, Westmead, NSW 2145 Australia; 2 School of Medical Sciences, Faculty of Medicine and Health, University of Sydney, Sydney, NSW 2006 Australia

**Keywords:** Notch1, Ankyrin, Heat flux, Mitochondria

## Abstract

Notch signalling pathway plays a key role in metazoan biology by contributing to resolution of binary decisions in the life cycle of cells during development. Outcomes such as proliferation/differentiation dichotomy are resolved by transcriptional remodelling that follows a switch from Notch^on^ to Notch^off^ state, characterised by dissociation of Notch intracellular domain (NICD) from DNA-bound RBPJ. Here we provide evidence that transitioning to the Notch^off^ state is regulated by heat flux, a phenomenon that aligns resolution of fate dichotomies to mitochondrial activity. A combination of phylogenetic analysis and computational biochemistry was utilised to disclose structural adaptations of Notch1 ankyrin domain that enabled function as a sensor of heat flux. We then employed DNA-based micro-thermography to measure heat flux during brain development, followed by analysis in vitro of the temperature-dependent behaviour of Notch1 in mouse neural progenitor cells. The structural capacity of NICD to operate as a thermodynamic sensor in metazoans stems from characteristic enrichment of charged acidic amino acids in β-hairpins of the ankyrin domain that amplify destabilising inter-residue electrostatic interactions and render the domain thermolabile. The instability emerges upon mitochondrial activity which raises the perinuclear and nuclear temperatures to 50 °C and 39 °C, respectively, leading to destabilization of Notch1 transcriptional complex and transitioning to the Notch^off^ state. Notch1 functions a metazoan thermodynamic sensor that is switched on by intercellular contacts, inputs heat flux as a proxy for mitochondrial activity in the Notch^on^ state via the ankyrin domain and is eventually switched off in a temperature-dependent manner.

**Video abstract**

**Supplementary Information:**

The online version contains supplementary material available at 10.1186/s12964-022-00886-4.

## Background

Notch signalling pathway is a metazoan novelty [1], whereby downstream signals contribute to coordinated resolution of diverse fate dichotomies confronted by individual cells via input from neighbouring cells. Decisions such as proliferation versus differentiation [2], or assumption of mutually exclusive sub-lineage fates [3] are modulated by Notch-mediated transcriptional remodelling of cycling cells [4]. Assumption of an alternative fate requires emergence of a competing fate and simultaneous abolition of the dominant fate, in a stepwise manner. Initially, Notch signalling pathway is switched on by *trans*- [4] or *cis*-ligands [5] that trigger enzymatic release of Notch intracellular domain (NICD) followed by subsequent translocation of NICD to the nucleus where it  associates with RBPJ. Transcriptional remodelling in the Notch^on^ state facilitates partial emergence of the transcriptional profile of a coupled competing fate (e.g. differentiation profile in the context of cycling cells) leading to a bistable transcriptional profile [6]. Subsequent transitioning to mono-stability by termination of the dominant fate and adoption of the competing fate requires a second wave of transcriptional remodelling that is invoked by switching from a Notch^on^ to Notch^off^ state [2, 3]. Both activation and inactivation of Notch signalling pathway are controlled at multiple levels.

While activation of Notch signalling has been extensively studied, molecular mechanisms for switching off the signalling cascade are not as well-defined. In the Notch^on^ state, termination of Notch signalling output requires intervention at four distinct levels: transcriptional silencing of the active notch locus, degradation of the available notch mRNAs, removal or inhibition of the existent Notch receptors, and dissociation of NICD from the binding partner RBPJ. Several mechanisms have been proposed for removal of the existing Notch receptors including endocytosis [7], autophagy [8] and ubiquitination [9]. As a membrane-tethered receptor, Notch requires endocytosis, not only to release the NICD from the membrane [10] but also to partition the receptor into late endosomes for degradation [7]. Apart from endocytosis, Notch can be degraded by incorporation into autophagosomes [8]. Finally, ubiquitination serves as another important decision point in regulating the half-life and activity of the membrane-bound Notch receptor. While mono-ubiquitination directs the receptor to the vesicular trafficking pathway, poly-ubiquitination leads to its degradation [11]. It appears that the post-translational modifications described above either fine-tune the steady-state rate of signalling output in the Notch^on^ state or set an upper limit to the signalling output by blocking further generation of NICD. To be effective, the post-translational regulation of Notch receptor must be complemented by transcriptional and post-transcriptional modulation of the encoding locus and the associated mRNAs.

We recently reported that transcription from human notch-1 locus is regulated by generation of a *cis*-natural antisense transcript that restricts the active transcriptional window to G0/early G1 phase of cell cycle [12]. The antisense transcript also regulates the availability of notch-1 transcript at G1 phase of cell cycle by calibrating RNA editing of the transcript and its subsequent degradation by nonsense-mediated decay [12]. The critical role of mRNA titre in determining Notch signalling output [12] is foreshadowed by the evolution of multiple parallel mechanisms that enhance the fidelity of notch transcriptional landscape [13, 14]. While knowledge of the Notch signalling cascade has grown extensively over the past decade [4], one major question remains unanswered; that is the mechanism(s) that regulate termination of Notch/RBPJ signalling output by triggering dissociation of the active transcriptional complex. A preliminary clue regarding transitioning from Notch^on^ to Notch^off^ state was provided by the observation that amplified mitochondrial activity during brain development coincides with abrupt inactivation of Notch signalling cascade and accelerated adoption of neuronal fate [15]. However, it remained unclear whether these two events are causally linked.

Here, we adopt an evolutionary approach to probe the causal link between mitochondrial activity and transitioning to the Notch^off^ state. We provide evidence that the ankyrin domain of Notch-1 has progressively evolved to acquire structural properties of a thermodynamic sensor that inputs heat flux as a proxy for mitochondrial activity in the Notch^on^ state and is eventually switched off in a temperature-dependent manner. This mechanism ensures optimal timing of major biological decisions, such as differentiation, by aligning the resolution of fate dichotomies to mitochondrial activity. Together with previous knowledge regarding transitioning from Notch^on^ to Notch^off^ state, our findings advance the blueprint of a circuitry that regulates biological decision making.

## Results

### Ankyrin domain as a stress sensor in major kingdoms of life

Notch is a transmembrane protein with an extracellular domain consisting of multiple epidermal growth factor-like (EGF) repeats, and an intracellular domain comprising a RAM domain and multiple tandem ankyrin (ANK) repeats [16]. Subsequent to enzymatic cleavage the intracellular domain (NICD) translocates to the nucleus, wherein the RAM and ankyrin domains of NICD associate with the ß-trefoil and C-terminal domains of a DNA-bound RBPJ (alias CSL) and MAML-1 to recruit RNAP-II [17, 18] and to *trans*-activate the downstream loci (i.e. Notch^on^ state). To disclose the mechanistic basis for transitioning from the Notch^on^ to the Notch^off^ state, we investigated the evolutionary basis for co-opting various domains of Notch receptors. We began by reconstructing the comparative network topologies populated by EGF and ANK domains of the representative Notch-1 receptor after the three-way split of plants, animals and fungi. The reconstruction was restricted to the best studied species from each taxonomic division; *Saccharomyces cerevisiae* from the fungi, *Arabidopsis thaliana* from the plant kingdom, and *Homo sapiens* as the representative metazoan species.

**Fig. 1:**
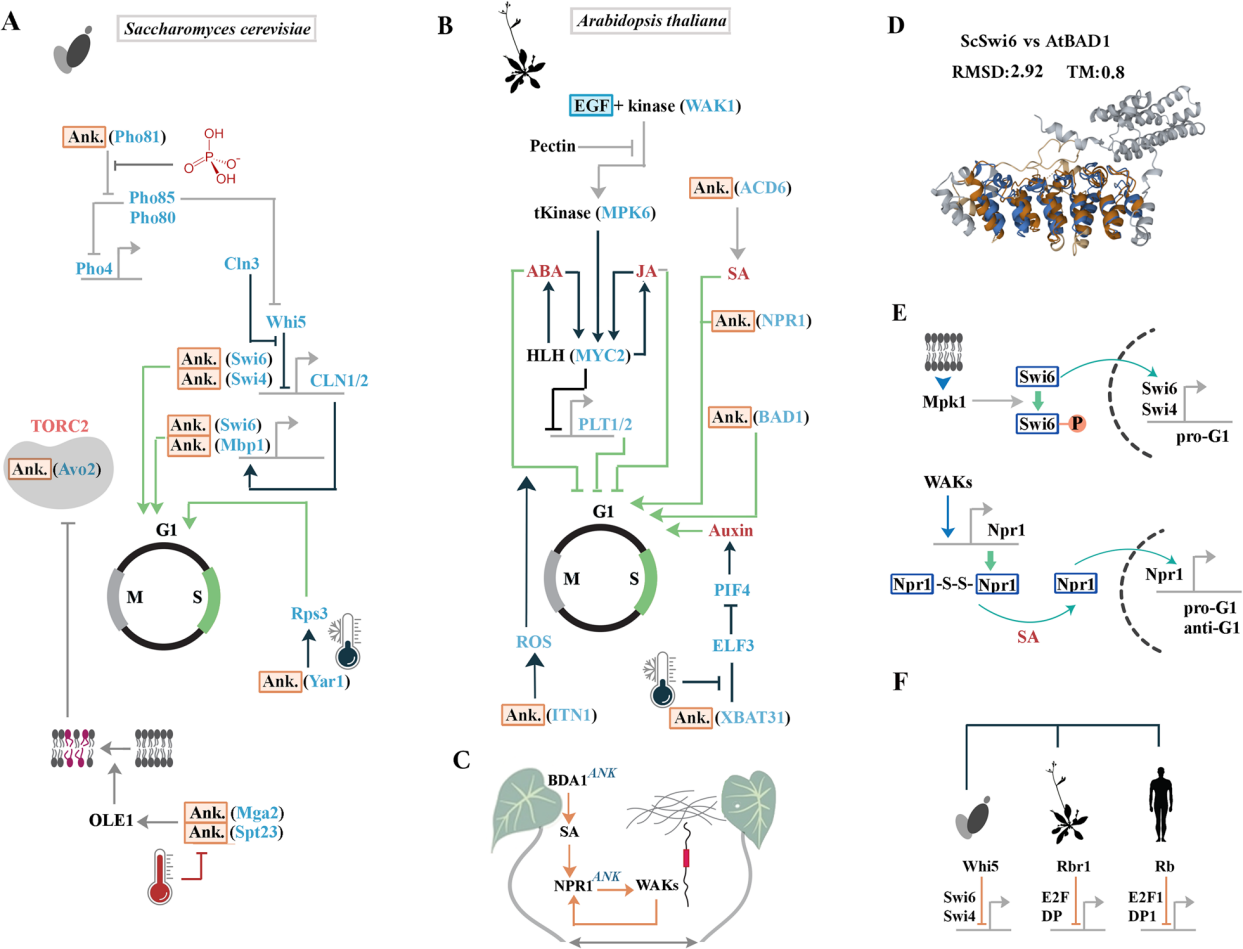
Network topology of cascades populated by ANK^+^ proteins. **a** ANK^+^ proteins in *S. cerevisiae* relay information regarding local stressors to cell cycle mediators via Swi6/Swi4 complex. Phosphate deprivation triggers activation of ANK^+^ Pho81 leading to inactivation of the Pho80-Pho85 complex and subsequent Pho4-dependent activation of phosphate-responsive genes. In phosphate-rich environment, Pho80-Pho85 complex inhibits the transcriptional repressor Whi5. ANK^+^ Avo2 is a plasma membrane stress reporter which is inhibited upon reduction of membrane tension [84]. ANK^+^ Mga2/Spt23 complex senses lipid-packing within the lipid bilayer [19] and activates Δ9 fatty acid desaturase of yeast, OLE1, at high temperature to enhance the fluidity and hence the mechanical durability of nuclear [20] and cytoplasmic membranes [21]. Finally, ANK^+^ Yar1 integrated environmental stressors (e.g. low temperature) into ribosome biogenesis [85]. **b** ANK + proteins in *A. thaliana* integrate local and systemic information regarding stressors to cell cycle of individual cells. **c** In *A. thaliana,* the ANK^+^ BDA1 binds to salicylic acid (stress hormone) leading to release of monomeric ANK^+^ NPR1. NPR1 upregulates the EGF^+^ Wall-associated kinases to heighten the response to local stressors that affect the cell wall. **d** Structural alignment of Swi6 and BDA1 reveals high homology of these ANK^+^ proteins. **e** While in yeast, adaptation to stressors is achieved by phosphorylation and exclusion of Swi6 from the nucleus, in plants stressors trigger translocation of Npr1 to the nucleus [86] and *trans*-activation of the downstream genes. **f** It is likely that partial uncoupling of Swi6 from cell cycle, facilitated by evolution of the E2F family of transcription factors in multicellular organisms, provided the opportunity for repurposing of ANK + proteins in transition to sessile multicellularity

While the EGF domain (InterPro ID: IPR001881) and the intrinsically disordered RAM domain are not deployed in *S. cerevisiae* (strain ATCC 204,508 / S288c), the ANK domain (InterPro ID: IPR002110) contributes to multiple related protein families. These ANK^+^ proteins facilitate adaptation of the yeast to exogenous stressors that affect the cell wall and plasma membrane (Fig. 1a). For example, ANK^+^ Mga2/Spt23 complex senses lipid-packing within the lipid bilayer [19] and activates Δ9 fatty acid desaturase of yeast, OLE1, at high temperature to enhance the fluidity and hence the mechanical durability of nuclear [20] and cytoplasmic membranes [21]. A key facet of accommodating stressors is modulation of cell cycle dynamics that is achieved by two ANK^+^ protein assemblies of yeast, namely SBF (Swi6/Swi4) and MBF (Swi6/Mbp1) complexes [22]. To adapt to stressors, Swi6 receives input from Mpk-1 protein kinase regarding cell wall integrity of yeast [23, 24] and reprograms cycle dynamics accordingly [25]. While ANK^+^ proteins remained the centrepiece of adaptation to stressors in multicellular life forms, novelties came about that enabled heightened response to stressors by integration of organism-level (i.e. systemic) memory of exposure to stressors. One such major change was recruitment of the EGF domain.

In plants, the EGF domain uniquely characterises a single protein family, wall-associated kinases (WAKs) (Fig. 1b). WAKs bind to cell wall pectin and activate stress adaptation pathways upon sensing alterations of cell wall by biotic and abiotic stressors [26]. *Trans*-activation of WAKs by the plant stress hormone salicylic acid (SA) [27] requires critical input from two ANK^+^ proteins, the upstream mediator BDA1 [28] and the downstream mediator NPR1 [27] (Fig. 1b). Therefore, WAKs interpret modifications of cell wall in the context of hormonal input from other, distal cells (Fig. 1c), a phenomenon that maximises the chance of survival [29] by calibrating the response of individual cells to systemic memory of exposure to stressors [30]. It becomes apparent that ANK^+^ proteins of fungi and plants, with high structural and functional homology (Fig. 1d), have populated key nodes of signalling cascades that facilitate adaptation to stressors by signalling input from cell wall integrity receptors (Fig. 1c, e).

**Fig. 2:**
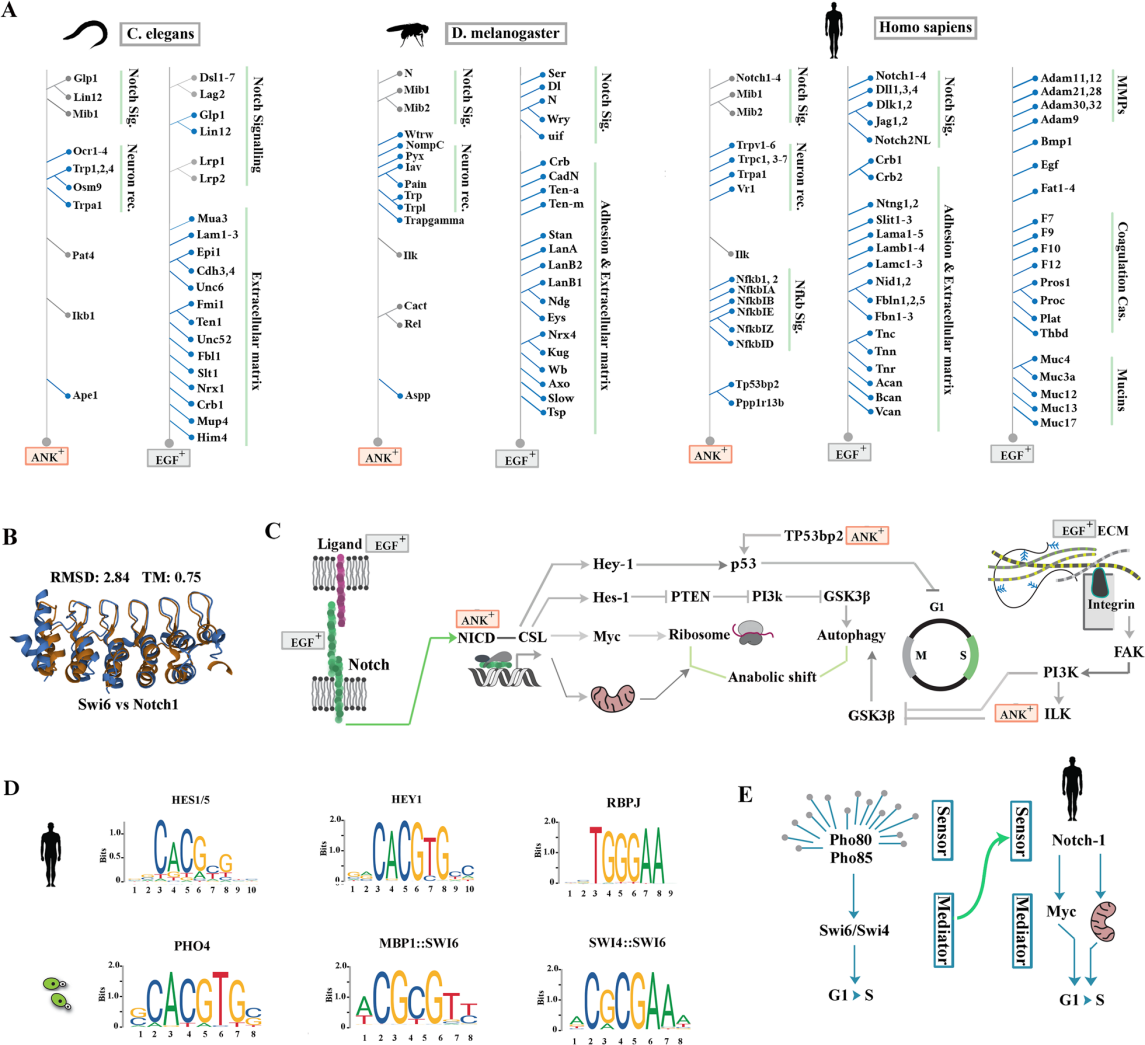
Network topology of cascades populated by ANK^+^ proteins in metazoan animals. **a** Restricted deployment of ANK^+^ and EGF^+^ domains in proteins responding to abiotic stressors in *C. elegans*, *D. melanogaster*, and human. **b** Structural alignment of Swi6 and Notch-1 ankyrin (1SW6 vs. 1YYH) reveals high homology of these ANK^+^ proteins. **c** The Notch receptor belongs to a major group of ANK^+^ proteins occupying critical nodes of a signalling hub that amplifies pro-anabolic activity by enhancing mitochondrial output, *trans*-activating the ribosomal loci, and by repressing autophagic flux. Simultaneously, the cascade prevents premature progression from G1 to S phase. **d** DNA sequences recognised by cell cycle regulators of *S. cerevisiae* and human Notch pathway mediators (database: JASPAR 2022). **e** Altered hierarchy of deployment of ankyrin domain in Swi6 and Notch signalling pathways as a downstream mediator of stress response and an upstream reporter of stressors, respectively

In metazoans, replacement of the yeast Swi6/Mbp1/CDC28 signalling axis [31] by the E2F family of transcription factors [32] (Fig. 1f), and the disappearance of cell wall facilitated repurposing and broader exaptation of EGF and ANK motifs by diverse protein families (Fig. 2a). In *C. elegans*, ANK domain characterises five major families, I. Notch homologues Glp1 and Lin12, and E3 ubiquitin ligase Mib1, II. Neuronal mechanosensory receptors, III. Integrin-linked kinase homologue Pat4, IV. Ape1 (a homologue of human Tp53bp2), V. The IκB homolog Ikb1 (Fig. 2a). This ANK^+^ cluster expands to include multiple components of the NF-κB signalling cascade in higher metazoans. The EGF domain in *C. elegans* is restricted to members of the Notch signalling pathway and structural components of the extracellular matrix, such as laminins. Similar to ANK, exaptation of EGF increases in higher metazoans where the domain contributes to other novelties such as coagulation factors and mucins of saliva (Fig. 2a). Ankyrin domain in metazoan animals is also deployed as a reporter of abiotic stress.

In neuronal ion channels ANK functions as a reporter of abiotic stressors in a rather unique manner. Modularity of ANK and spatial organisation of individual repeat modules gives rise to a molecular spring which stores and releases mechanical energy [33]. This structural property has been utilised in neuronal ion channels to generate a thermal nano-spring [34] that undergoes temperature-dependent structural changes leading to channel opening [35]. In higher metazoan species with a closed circulation [36], a reduction of oxygen partial pressure in capillaries is directly sensed by the Band 3–ankyrin complex of RBCs leading to increased deformability of RBCs, increased velocity of blood flow, and re-establishment of the oxygen tension [37]. Likewise, function of the key ANK^+^ proinflammatory transcription factor NFκB is regulated by oxidative stress [38] and thermal fluctuation [39].

Apart from high structural homology of the ankyrin domains (Fig. 2b), the function of Notch-1 in driving progression of cell cycle (G1-S transition) by regulating the anabolic flux bears close similarity to Swi6 (Fig. 2c, d). There is, however, a radical difference in the network topologies and the signalling logics of the Swi6 and Notch1 pathways (Fig. 2e). Swi6 functions as a downstream mediator of Pho80-Pho85 family of cyclin-dependent kinases that interact to report abiotic stressors. Transition to multicellularity was concurrent with redundancy of the Pho80-Pho85 pathway [40] leading to emergence of Notch-1 as an upstream pro-anabolic mediator, a hierarchical position that must be occupied by a protein with sensory capacity (Fig. 2e). Given the sensory functionality of the ankyrin domain in other proteins as discussed above, we postulated that deployment of the ankyrin domain could have equipped Notch-1 with a capacity to interact with and report specific stressors. To investigate whether Notch ankyrin domain could interact with stressors, we probed evolutionary adaptations of ANK after deployment of the domain as a structural component of the Notch receptor with reference to Swi6.

### ANK domain of Notch-1 has adopted structural features that renders it responsive to thermal fluctuations

**Fig. 3:**
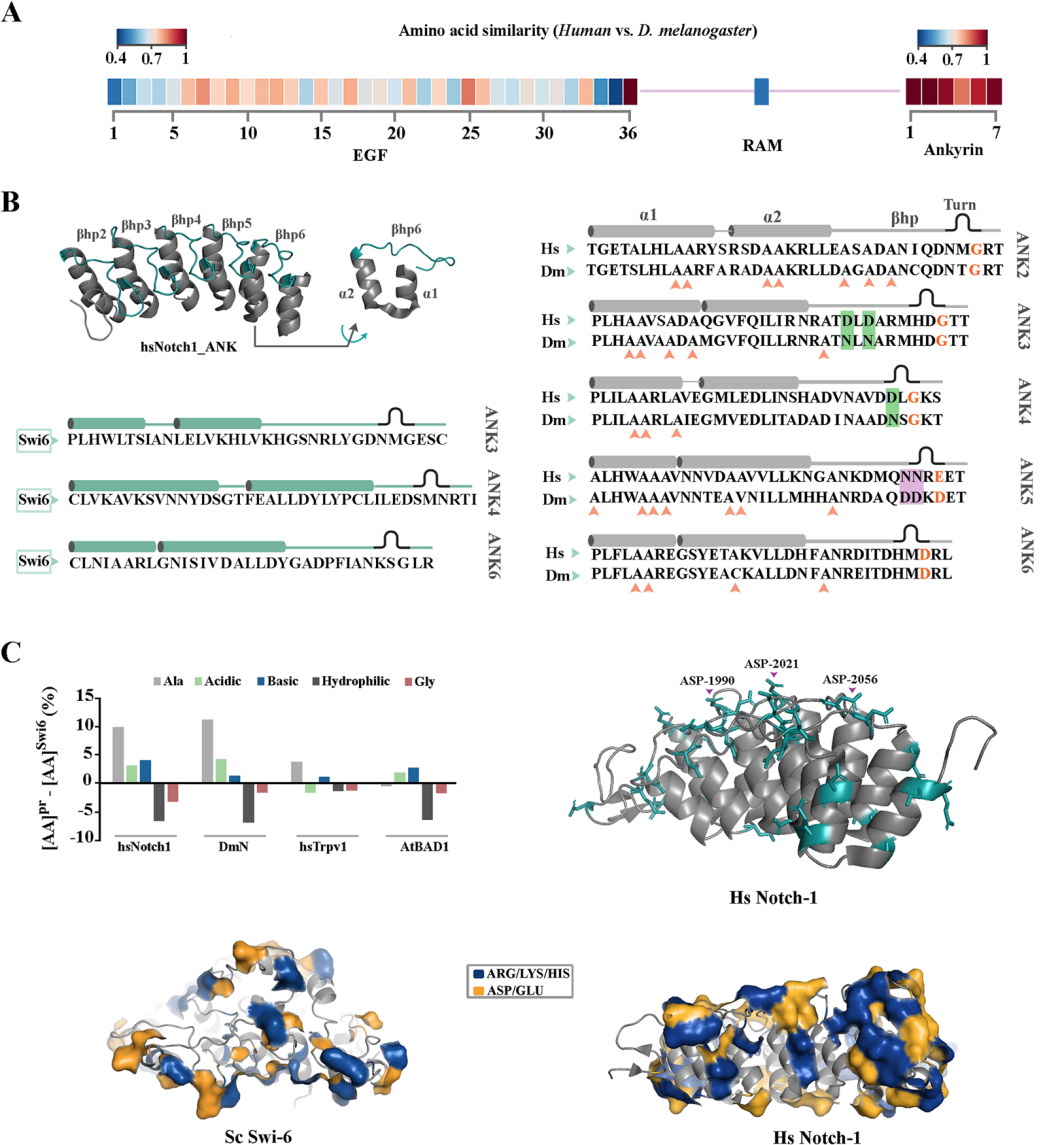
Uncoupled evolution of Notch-1 ankyrin domain from other functional domains of the receptor. **a** Alignment of the primary sequence of EGF, RAM, and ANK domains of human Notch-1 and Drosophila N reveals high conservation of ANK domain despite significant divergence of the EGF and RAM domains (See Additional file 1: Fig. S1). **b, c** Comparison of the primary sequence of human Notch-1 (hs-Notch1) and *D. melanogaster* N (Dm-N) to *S. cerevisiae* Swi-6 shows marked enrichment of alanine, with the highest propensity for helix formation (42), in α-helices of Notch ankyrin domain. A second important structural adaption of Notch ankyrin domain was enrichment of acidic amino acids with negative side chains in solvent exposed surface of α-helices and β-hairpins

Despite significant inter-species divergence of ligand-binding EGF repeats and the RBPJ-binding RAM domain of Notch (amino acid similarity < 75% except for EGF-32), the ANK domain of the protein is highly conserved between the human and fruit fly orthologues (Fig. 3a, amino acid similarity > 75%). Absence of a significant divergence suggested that the ANK domain of Notch is subject to selective pressures that are common to both species, and that are somewhat uncoupled from the selective pressures that have shaped the evolutionary trajectories of the EGF and RAM domains. To gain insight into the potential selective pressure acting upon ANK domains of the Notch protein, we mapped structural adaptations of the domains with reference to yeast Swi6.

**Fig. 4:**
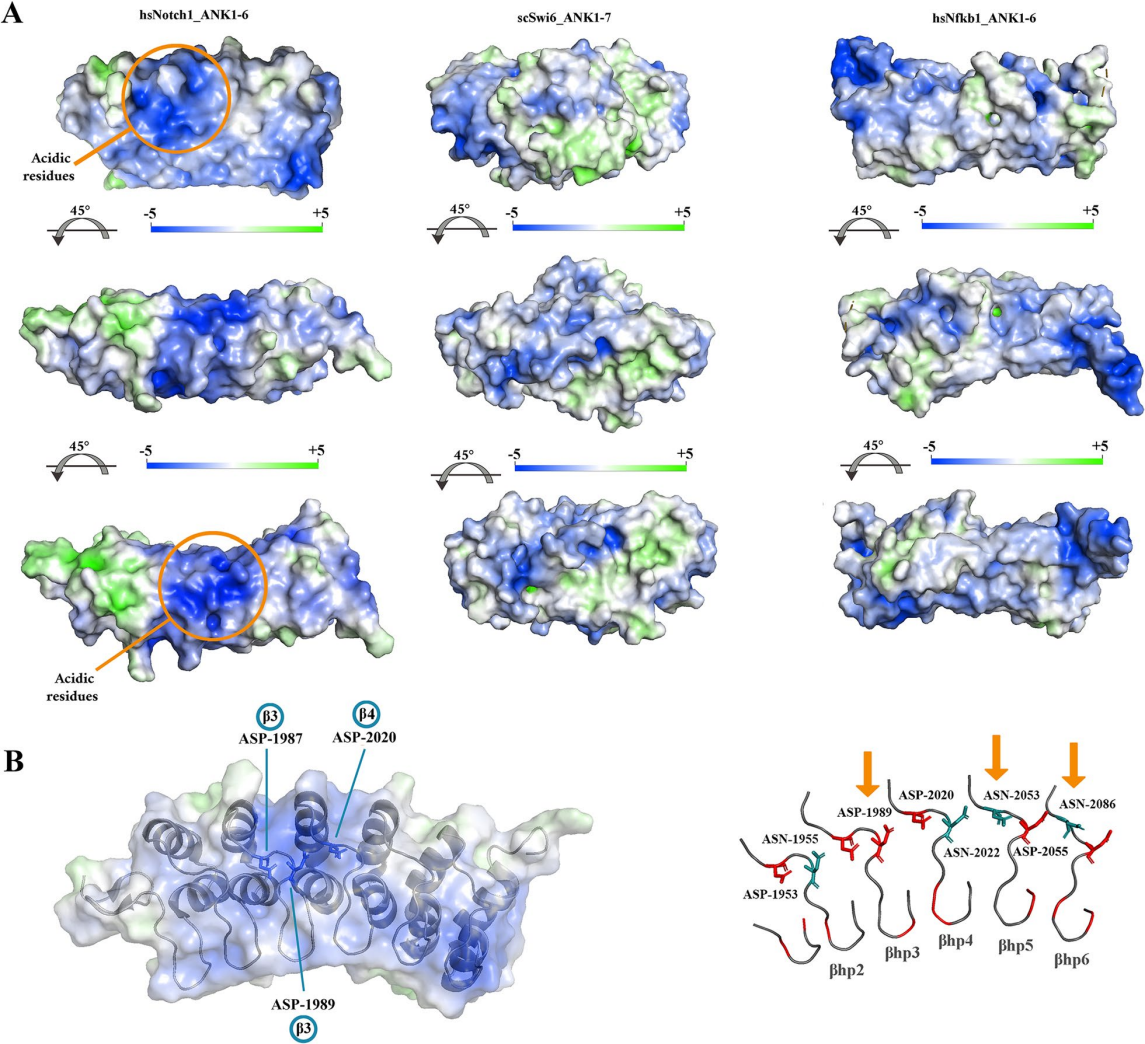
Electrostatic surface potential of Notch-1 ankyrin domain reflects the enrichment of acidic residues. **a** The electrostatic surface potential values for Notch-1 ankyrin (PDB: 1YYH), Swi6 (PDB: 1SW6), and NF-κB (PDB: 1IKN), calculated using Adaptive Poisson-Boltzmann Solver (APBS), are here reported. Surface potentials are on a [− 5,5] blue-white-green colour scale (kJ.mol^−1^.e^−1^). The top and the bottom panels provide a surface view of the β-hairpins and α-helices of the studied molecules, respectively. **b** The key acidic residues that populate the negatively charged pocket of the β-hairpins are highlighted in stick mode and labelled accordingly. The right cartoon shows a simplified view of the β-hairpins (βhp1-6) of human Notch-1 with acidic and basic residues highlighted in red and turquoise, respectively. Arrows point to two major evolutionary adaptations in β-hairpins of Notch-1 ankyrin domain, I. Substitution of ASN-1989 by ASP-1989 (refer to ANK4 of *D. melanogaster* as per Fig. 3b), and II. a reversal of the spatial order of ASP-ASN in ANK5 and ANK6 that enhances the anisotropic charge distribution

Each module of ANK domain in Notch is comprised of 33 residues that form two anti-parallel α-helices (α1 and α2) followed by a β-hairpin (βhp) (Fig. 3b). Structural alignment of the consensus ANK sequence revealed major adaptations in ANK domains of the Notch protein. A key structural adaptation was enrichment of alanine and charged residues in α-helices and β-hairpins of the ANK domains of the human Notch-1 receptor which came at the cost of depletion of hydrophilic residues (Fig. 3c). Alanine is a known stabiliser of α-helices [41, 42], a propensity that is amplified by proximity to charged residues [43]. We used a PDB structure with a resolution better than 1.55 Å (ID: 2F8Y) to study spatial distribution of the charged residues. While the side chains of positively charged residues mainly populated the pocket formed between the inner α-helix (i.e. α2) and the β-hairpins (RBP-J binding pocket), negatively charged side chains occupied the outer surface of the outer α-helices (i.e. α1) and the β-hairpins (Fig. 3c). It is known that arrangement of side chains with similar electrostatic charges in dense pockets could potentially lead to coulomb repulsion [44]. Application of the Adaptive Poisson-Boltzmann Solver (APBS) to visualise the electrostatic potential around Notch-1 ANK revealed two zones of high net negative potential (Fig. 4a). One region (≈289 Å^2^) corresponded to the solvent-exposed surface of the β-hairpins of ANK3 and ANK4 (residues ASP-1987, ASP-1989, ASP-2020) (Fig. 4b). The other zone (≈327 Å^2^) was populated by the side chains of the acidic residues in α2-helices of ANK4 (residues GLU-2008, GLU-2012, ASP-2013). Anisotropic distribution of dense pockets of similar charges in Notch ANK domain was a unique structural adaptation that was not observed in Swi6 (Fig. 4a) and in other studied ANK domains such as NF-κB (Fig. 4a). The combination of high net charge and reduced hydrophobicity is a unique structural feature of "natively unfolded" proteins [45] and localised adoption of this feature by ANK4 (Fig. 4b) is expected to destabilise the ankyrin domain of Notch protein. This finding raised an important question. Is Notch ankyrin domain unique in adopting the described destabilising structural feature?

**Fig. 5:**
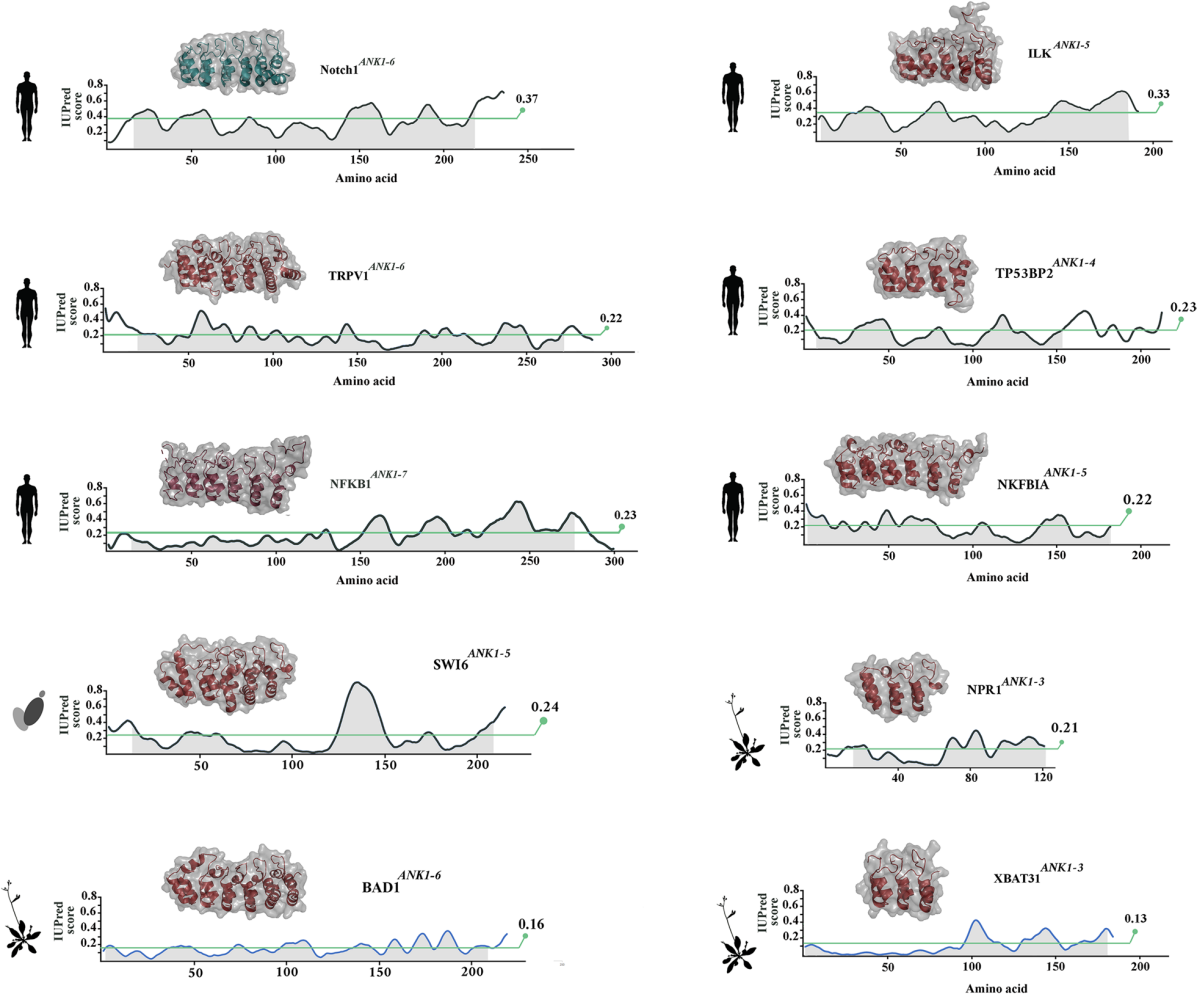
Divergent evolution of disorder in Notch ankyrin domain of eukaryotes. Plots show IUPred score for representative ankyrin domains from various families in human, *S. cerevisiae*, and *A. thaliana* (horizontal line: average IUPred score)

We used IUPred [46] to gain quantitative insight into divergence of Notch1 ankyrin domain from ankyrin domains of other proteins, in adopting destabilising structural features (Fig. 5). Consistent with previous analyses, Notch-1 ankyrin domain exhibited a high IUPred score (average: 0.37). The average IUPred score for ankyrin domains of other human proteins was 0.22, a value close to the average IUPred score of *S. cerevisiae* ankyrin domains (average score: 0.21). Ankyrin domains of *A. thaliana*, on the other hand, showed the opposite trend of a reduced IUPred score (Average score: 0.16). We then estimated the entropic cost of Notch-1 structural adaptation knowing that the IUPred score is generated by a method developed by Thomas and Dill which uses Boltzmann statistics to extract the dimensionless energy-like inter-residue interaction potential [47]:

$${E}_{ij}=\frac{energy}{kT}=-\mathrm{ln}(\frac{{\rho }_{ij}}{{\rho }_{ij}^{*}})$$

where k is the Boltzmann constant, and T is absolute temperature, and ρ_ij_ and ρ^*^_ij_ represent the pairing frequencies of amino acid type i with amino acid type j in a protein of interest and in a reference state, respectively. After rearrangement of the latter equation, n_A_kΔT × (IUPred^notch^/IUPred^ref^) corresponds to the entropic cost of the evolutionary adaptations of Notch-1 ankyrin domain (n_A_: Avogadro’s number, IUPred^notch^: IUPred score for Notch-1 ANK, IUPred^ref^: average IUPred score for ANK domains of other human proteins). This corresponds to an entropic cost of

$$\begin{aligned} {\text{Entropic}}\;{\text{cost }} &= { 3}.{5}\Delta {\text{T }}({\text{cal}} \cdot {\text{mol}}^{{ - {1}}} ) \\ &\times { 23}0 \, \left( {{\text{residues}}\;{\text{in}}\;{\text{ANK}}} \right) \\ &= 0.{8}\Delta {\text{T }}({\text{kcal}} \cdot {\text{mol}}^{{ - {1}}} ) \end{aligned}$$

In other words, Notch ankyrin becomes destabilised by 0.8ΔT (kcal⋅mol^−1^) in a temperature-dependent manner and relative to the more stable ANK domains of other proteins. Given the ΔG^0^ for binding of Notch1 intracellular domain to RBPJ (≈ -8 kcal⋅mol^−1^) [48], a temperature rise of 10°K could potentially provide sufficient energy to disrupt the association of Notch1 intracellular domain and RBPJ. Further, given the reported flexibility of ankyrin domain along one major axis that accommodates the flexible β-hairpins [34], even a more modest temperature rise of ≈3°K (i.e. 10°K/3 degrees of freedom) would be theoretically sufficient to trigger dissociation of the Notch-1/RBPJ/MAML complex. In order to investigate the behaviour of Notch-1 ankyrin domain as a function of temperature, we carried out molecular dynamic simulations at different temperatures.

**Fig. 6:**
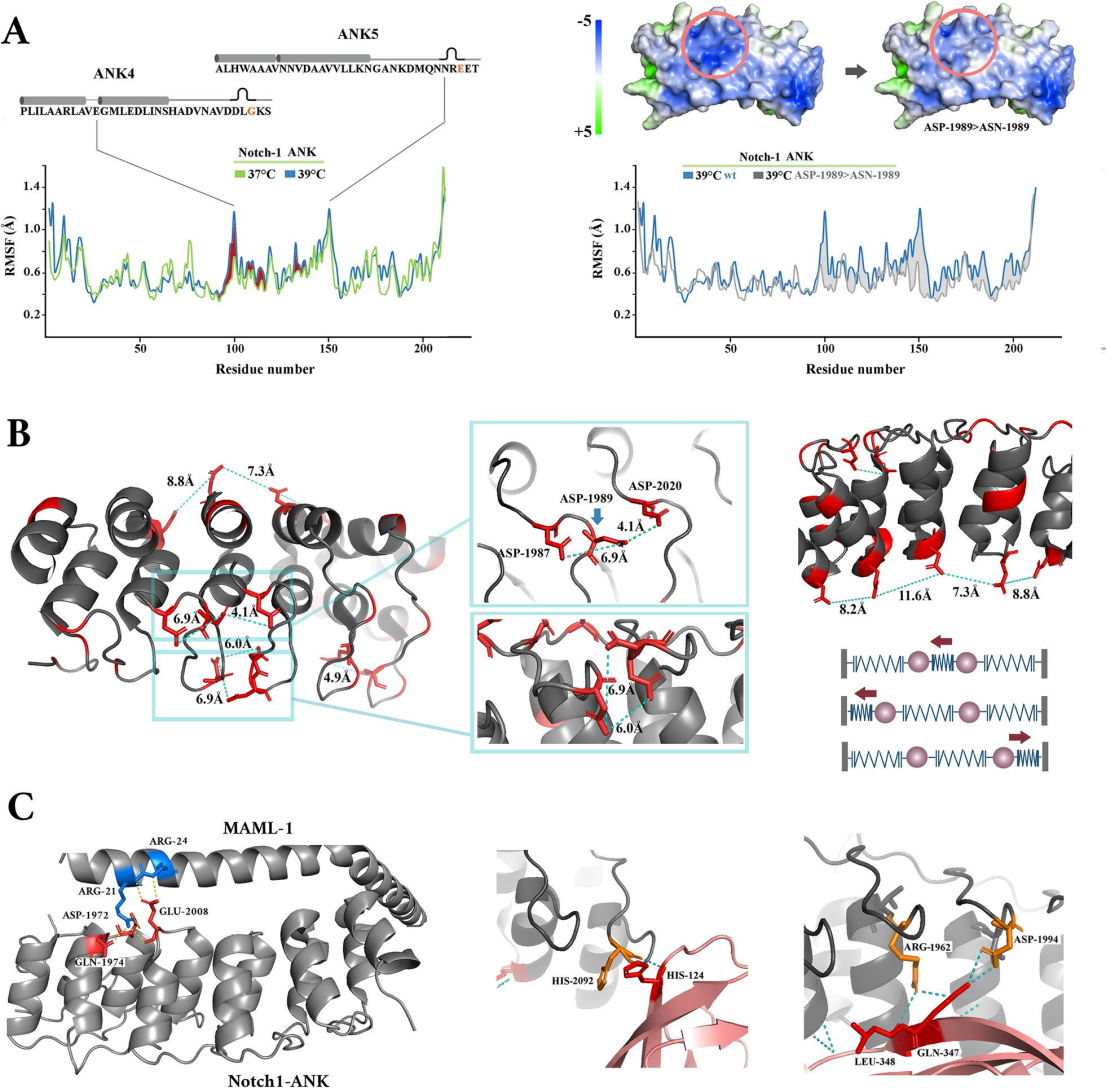
Analysis of the temperature-dependent structural fluctuations of Notch-1 ankyrin domain. **a** The left plot shows Root mean-square fluctuation **(**RMSF) of alpha carbon (Cα) of Notch ANK (Met-1872: Asp-2114) at 37 °C and 39 °C. The right plot shows RMSF after substitution of ASP-1989 > ASN-1989. **b** Spatial organisation of acidic residues and the distance between side chain carboxyl groups of these amino acids (PDB: 2F8Y). The structural arrangement of acidic residues could give rise to coulomb repulsion and translational vibration of ANKs (particularly ANK4) along the long axis of the domain in a manner akin to multiple coupled oscillators (bottom right schematic). **c** Hydrogen bonds between MAML-1, Notch-1 ankyrin domain, and RBPJ in the active DNA-bound transcriptional complex (PDB: 2F8X)

Molecular dynamics analysis of Notch-1 ankyrin was performed on a PDB structure with a resolution of 1.55 Å (ID: 2F8Y) (see methods for the details). The root-mean-square fluctuation (RMSF) that represents fluctuation of individual amino acids during the trajectory of the dynamics, revealed that two regions fluctuate more than others (Fig. 6a). A region corresponding to the negatively charged pocket formed by ARG-2060, GLU-2061, and GLU2062 of ANK5 was unstable both at 37 °C and 39 °C. A second region corresponding to the negatively charged zone populated by the side chains of the acidic residues in α2-helices of ANK4 (residues GLU-2008, GLU-2012, ASP-2013) became unstable only at 39 °C. Both these regions exhibited high net negative potential suggesting that coulomb repulsion is potentially involved in driving the fluctuations. Consistent with this notion, mutating ASP-1989 to ASN-1989 in silico, to reduce the repulsive force from ASP-1987 and ASP-2020 on ANK4, resulted in dampening of molecular fluctuations at 39 °C (Fig. 6b). The *in-silico* findings regarding the critical role of ASP-1987 were aligned to the observation that the substitution in Adams-Oliver syndrome leads to disruption of Notch-1 signalling [49]. The acidic amino acids in ANK4, apart from fluctuating in a temperature-dependent manner, are essential for stabilising the RBPJ/Notch1/MAML1 transcriptional complex (Fig. 6c). GLU-2008 of ANK4 and ASP-1972 of ANK3 establish five hydrogen bonds with MAML-1 (total number of inter-chain hydrogen bonds: 8) (Fig. 6c). Further, ASP-1994 of ANK4 makes two hydrogen bonds with GLN-347 of RBPJ (Fig. 6c). The dual role of the acidic amino acids of ANK4 in stabilising and destabilising the Notch1 transcriptional complex bolstered the suggestion that the ankyrin domain has evolved to function as a thermodynamics sensor that terminates Notch signalling output in a temperature-dependent manner. Accordingly, enrichment of alanine and the resultant stabilisation of α-helices [41–43], is a structural adaptation that enhances efficient transmission of vibrational energy via rigid α-helices as opposed to its dissipation as heat [50, 51]. We finally set about to experimentally test whether increased temperature would result in termination of Notch1 signalling output by destabilisation of ankyrin domain and whether such an increased temperature arises upon cannibalisation of erythroblasts and the resultant activation of mitochondria [15].

### Heat flux: a physical signal arising at the interface of metazoan biophysics and bioenergetics

**Fig. 7:**
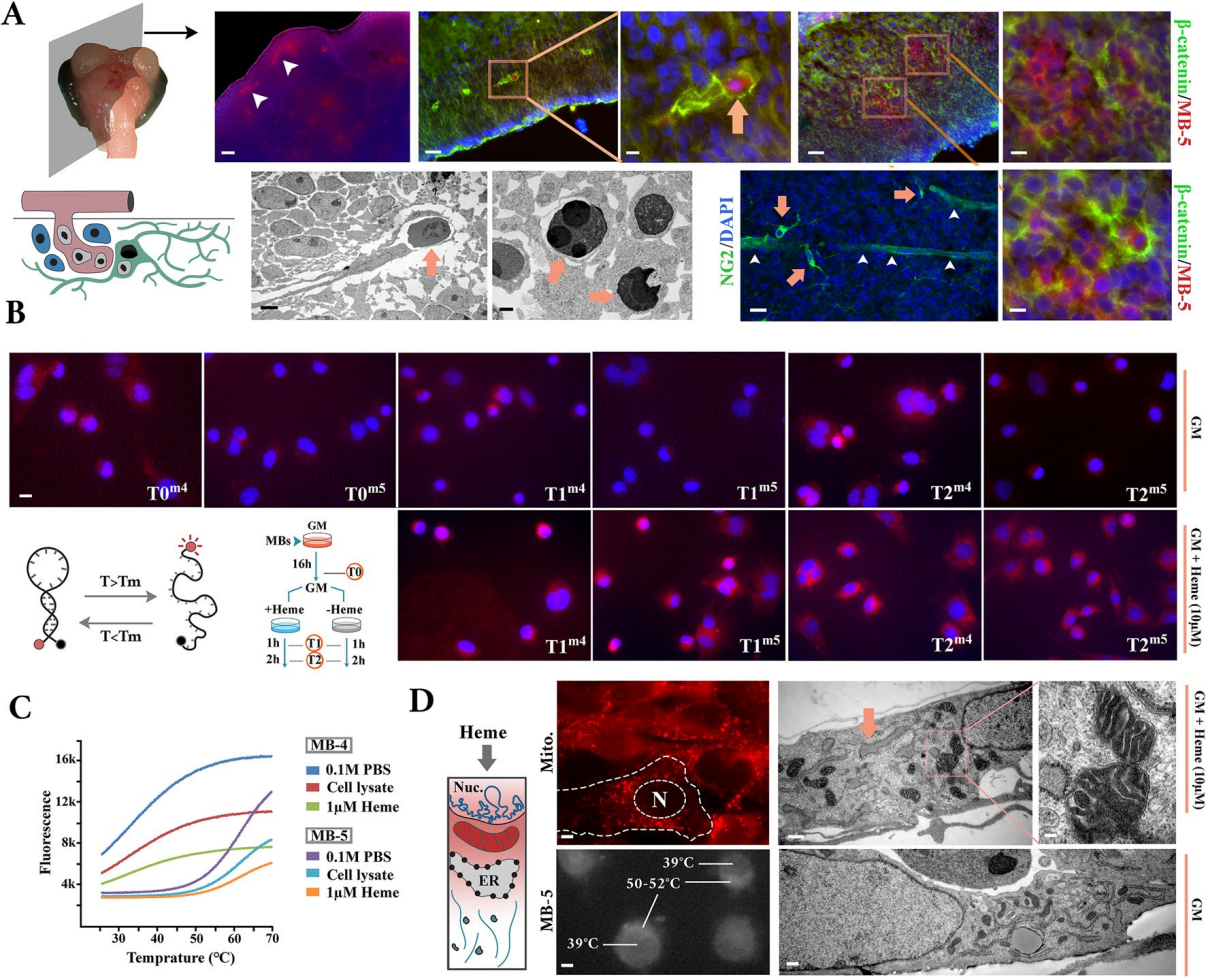
Crosstalk between mitochondria and the eukaryotic host using heat flux as a biophysical signal. **a** Application of molecular thermometers reveals internal temperature of developing chicken midbrain (HH stage 9–10) at single cell level. Electron micrographs show erythroblasts inside the blood vessels (left) and subsequent to cannibalisation (right). Top scale bars (from left to right in µm): [8, 10, 20, 30, 40], bottom scale bars (from left to right in µm) [4, 1.5, 15, 10]. **b** Micro-thermographic visualisation of cycling cells using molecular beacons (MB-4: m4, MB-5: m5) after addition of heme^−^ and heme^+^ growth medium to the cells (T1: 1 h, T2: 2 h after adding the medium). Reduced intensity of MB-5 from T1 to T2 could potentially be a consequence of inhibition of OXPHOS via generated ATP, depletion/saturation of intermediate metabolites of TCA cycle, or heat-dependent denaturation of unstable cytochrome c. Scale bar: 5 µm. **c** Temperature-dependent intensity of fluorescence signal (y-axis) in PBS, cell lysate, and heme/PBS as per methods. While cell lysate and heme reduce the intensity of signal, sensitivity of the molecular beacons and overall kinetics of temperature-dependent activation remain unaffected. The reduced intensity of MB in cell lysate results from absorption of thermal energy by various proteins, and nucleic acids. Further, the emitted photons can potentially be absorbed by various proteins (including heme-proteins). In heme-treated samples, the reported capacity of heme to harvest the emitted light photons reduces the intensity of MBs. **d** Electron micrographs (right) show perinuclear localisation of mitochondria separated from the rest of cytoplasm by an intermediate zone occupied by dilated endoplasmic reticulum. The active mitochondria with enhanced membrane potential (top left; Mito.: MitoTracker red, control heme^−^ samples: Additional file 1: Fig. S2) increase the perinuclear and nuclear temperature (bottom left). The slight variability in the intensity of molecular beacon reflects variability of electroporation. Top scale bars (from left to right in µm): [3, 1, 0.2], bottom scale bars (from left to right in µm): [1, 3]

In order to measure the intracellular temperature, we used two L-DNA (optical isomer of naturally occurring D-DNA) molecular beacons (MB) that do not bind to cellular nucleic acids and hence accurately report alterations of intracellular temperature [52]. The L-MB4 and L-MB5 had a T_m_ of 36 °C and 50 °C, respectively (see Methods). We employed *ex ovo* electroporation of MB4 and MB5 into chick embryos (Hamburger-Hamilton stage 9–10) to investigate the intracellular temperature of neuroepithelial cells upon cannibalisation of erythroblasts. In the mesencephalon, MB-5 reported an intracellular temperature of ≥ 50 °C in the intravascular erythroblasts prior to cannibalisation by neuroepithelial cells (Fig. 7a). This observation provided an internal control for the DNA-based micro-thermography as heat production is a reported feature of avian erythroblasts that is tuned by mitochondrial activity [53] whose temperature approaches 50 °C upon activation of oxidative phosphorylation [54]. Upon cannibalisation of erythroblasts, the intracellular temperature of cannibalistic cells also raised to ≥ 50 °C (Fig. 7a). While this observation provided assurance that the raised temperature is a physiological phenomenon that occurs during brain development, the presence of non-cannibalistic cells with a normal temperature and the transient nature of this event made it challenging to advance the findings in vivo. In parallel experiments in vitro, incubation of cultured neural progenitor cells in heme-rich medium (10 μM heme) confirmed that access to heme for t = 1 h is sufficient to raise the perinuclear temperature to ≥ 50 °C and the nuclear temperature to 39–40 °C in a synchronised manner and in the entire cellular population (Fig. 7b, c). This nuclear-cytoplasmic gradient is consistent with perinuclear clustering of active mitochondria (Fig. 7d) [55], and curbed diffusion of the thermal energy via the nuclear membrane that has a lower thermal conductivity compared to that of water (0.20 W.m^−1^.K^−1^ for lipid bilayer vs. 0.63 W.m^−1^.K^−1^ for water) [56]. Given that access to exogenous heme leads to suppression of the Notch-1 signalling pathway [15], we next asked whether increased temperature mediates this effect. To answer this question, cells were incubated at a temperature of 39 °C for 2 h to uniformly increase the cytoplasmic and nuclear temperatures to 39 °C. To improve the accuracy of control experiments, we carried out preliminary experiments to determine the incubation temperature at which the heat sink buffers against transient temperature rise beyond 37 °C due to mitochondrial activity. Based on the preliminary results, the control experiments were performed at 35 °C for 2 h.

**Fig. 8:**
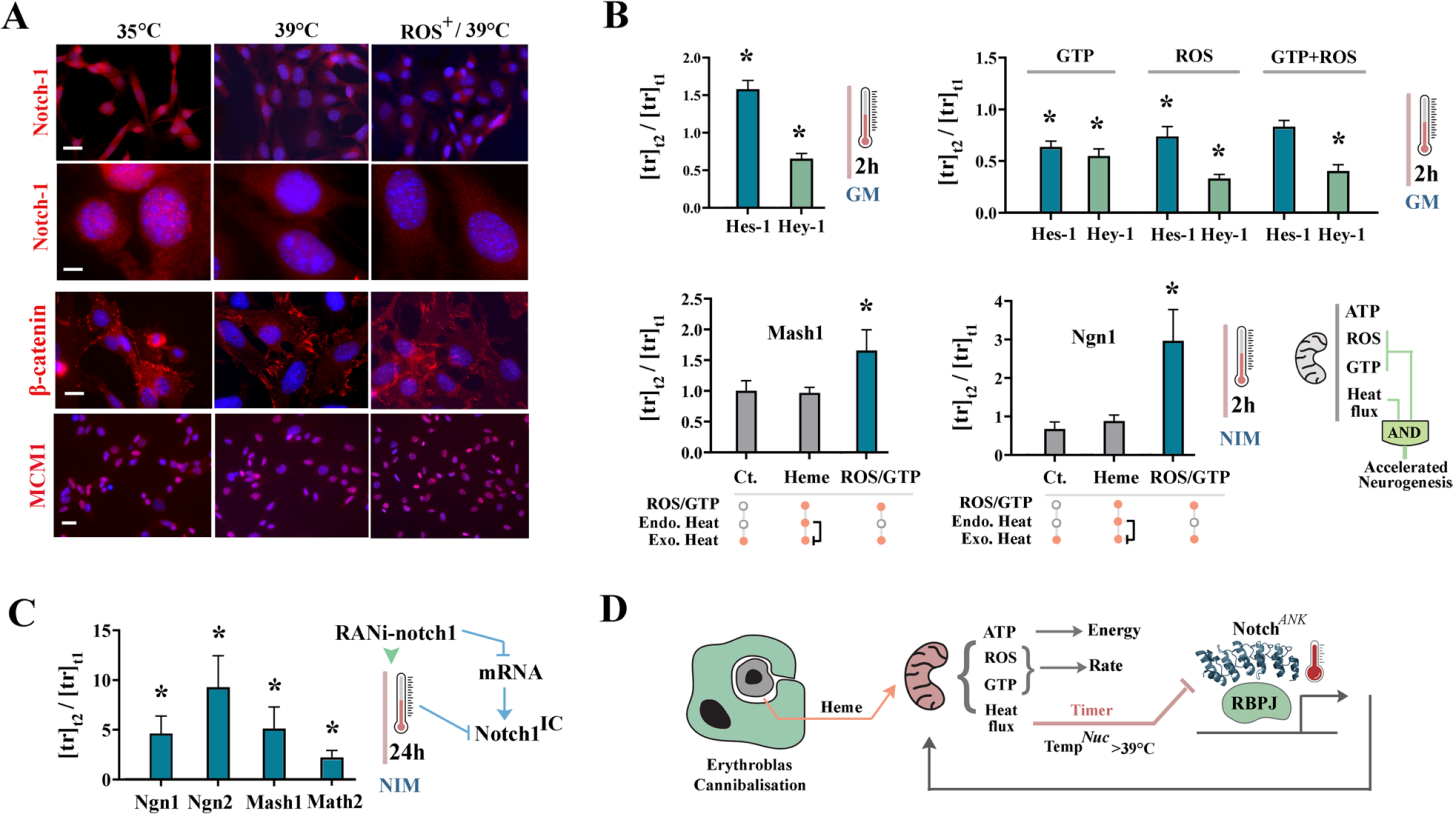
Heat flux links mitochondrial activity to differentiation of the host cell. **a** Immunohistochemical visualisation of intracellular Notch-1, β-catenin, and MCM1 in neural progenitor cells at 35 °C and 39 °C (ROS: H_2_O_2_) (Control micrographs: Additional file 1: Fig. S3). Scale bars (from top to bottom in µm): [5, 10, 20, 35]. **b** The top box plots show expression of Notch-1 downstream mediators after incubation of cells for 2 h at 35 °C (t_1_) and 39 °C (t_2_) in growth medium (GM). The bottom box plots show expression of pro-neural transcription factors after incubation of cells for 2 h at 35 °C (t_1_) and 39 °C (t_2_) in neural induction medium (NIM) (*p < 0.01). The schematic diagram (bottom right) shows topology of an “AND logic” gate that operates by input from mitochondrial ROS/GTP and heat flux to regulate the tempo of differentiation. **c** Expression of pro-neural transcription factors after RNAi-mediated depletion of notch-1 mRNA, incubation of 16 h in growth medium, and induction of differentiation by incubation in neural induction medium for 2 h at 35 °C (t_1_) and 39 °C (t_2_) (*p < 0.01). **d** Schematic image shows the proposed role of Notch-1 ankyrin as a thermodynamic sensor whereby Notch-1 pathway amplifies mitochondrial activity and the resultant thermal energy functions as negative feedback to recalibrate the activity of the signalling cascade. During cannibalisation of erythroblasts, access to copious heme and mitochondrial hyperactivation usurps this mechanism to supress Notch-1 and trigger accelerated differentiation of neural progenitor cells

Incubating the neural progenitor cells for 2 h at 39 °C, with or without H_2_O_2_ (a proxy for reactive oxygen species of mitochondrial origin), was sufficient to trigger depletion of the intracellular Notch-1 from the nucleus (Fig. 8a). Endogenous controls, the membrane-tethered β-catenin and the nuclear MCM1 (minichromosome complex 1), remained unaffected by the increased temperature. We next studied the impact of temperature on two downstream mediators of Notch-1 signalling. While the expression of Hey1 is specifically controlled by Notch-1, Hes-1 is regulated by Notch-dependent and Notch-independent mechanisms [57]. Therefore, Hes-1 provides an internal control for the specificity of the impact of temperature on Notch-1 signalling output. Downregulation of Hey-1 at 39 °C (Fig. 8b) supported the IHC findings regarding temperature-dependent removal of intracellular Notch-1 from the nucleus (Fig. 8a). Hes-1, on the other hand, was upregulated at 39 °C. The upregulation of Hes-1 suggests that the impact of temperature is somewhat specific to Notch-1 signalling output. Adding other mitochondrial products, GTP and ROS, did not alter the effect of temperature on Notch-1 downstream reporters (Fig. 8b). We finally questioned whether temperature-dependent downregulation of Notch-1 signalling output would accelerate neuronal differentiation. In heme-treated cells, pro-neural transcription factors Ngn-1 and Mash-1 were not differentially expressed at 35 °C and 39 °C. This is expected as addition of heme leads to amplified production of heat by mitochondria and hence obliterates the exogenous heat gradient. Mitochondrial by-products GTP and ROS, however, required exogenous heat to upregulate the level of Ngn-1 and Mash-1 (Fig. 8c). This finding was consistent with the demonstrated role of Notch-1 in maintaining stemness and in opposing neuronal differentiation [2]. Further, RNAi-mediated depletion of Notch-1 transcript complemented the impact of increased temperature on intracellular Notch-1 and led to more pronounced upregulation of the pro-neural transcription factors. Taken together, findings indicate that the heat produced by mitochondria is an essential biophysical signal, rather than a by-product, that operates in combination with GTP and ROS to regulate the rate of neuronal differentiation by supressing Notch-1 signalling output. As predicted by molecular dynamics of the ankyrin domain of Notch-1, elevation of the nuclear temperature to 39 °C (equivalent to a perinuclear temperature of 50 °C) was sufficient to terminate the signalling output of Notch-1 signalling cascade.

## Discussion

Herein, we combined an evolutionary approach with molecular biology to probe an unresolved facet of the Notch-1 signalling pathway, that is the mechanistic basis for termination of Notch-1 signalling during neuronal differentiation. We provide evidence that the ankyrin domain of Notch-1 has adopted specific structural features in order to function as a thermodynamic sensor that inputs heat flux as a proxy for mitochondrial activity in the Notch^on^ state and is eventually switched off in a temperature-dependent manner. Evolutionary adaptations that gave rise to this thermodynamic switch were facilitated by two major adaptations in multicellular metazoans.

The three-way split of plants, animals and fungi [58] was concurrent with altered signalling network topology of cell cycle in multicellular organisms. Not only did the E2F family of transcription factors, Rb, and various cyclins [32] replace the yeast Swi6/Mbp1/CDC28 signalling axis [31], but also the cell-intrinsic autonomy of cell cycle was partially abandoned to install a collective cycling mode [59]. Apart from cell cycle, the nature of outside-in stress signals capable of reprogramming the cell changed markedly in metazoan animals. For example, components of the cell wall integrity pathway [59] became partially redundant in metazoans due to the absence of a cell wall. Altered signalling network topology of cell cycle was a key step in facilitating the repurposing of proteins with ankyrin domains that originally functioned as stress sensors. While major groups of stress signals and receptors became redundant, it is noteworthy that transition to multicellularity gave rise to a new set of stress sensors.

The average cellular metabolic rate is estimated as ≈0.5 pW for bacteria, and as ≈2200 pW for eukaryotes [60]. The rate of heat production is higher by an order of magnitude during development. For example, zebrafish embryos at the 2-cell stage exchange heat at an approximate rate of 30 nW/cell [61]. Given the specific heat capacity of water (4.184 J⋅g^−1^⋅K^−1^) and the typical eukaryotic cell mass (≈1 ng), a conservative heat output of 20 nW/cell is expected to induce a temperature spike of ≈4.8 K in a period of one second (i.e. 20 nJ.s^−1^/4.184 nJ⋅ng^−1^⋅K^−1^ × 1 ng). This estimated value is consistent with the experimentally measured temperature spike of ≈4.8 K/s that is observed upon near-complete conversion of mitochondrial output to heat [62]. The generated heat has to dissipate and biophysical parameters that constrain the dissipation of thermal energy put an upper limit to metabolic rate even in unicellular organisms [63]. In a closely associated population of cells dissipation of heat by convection becomes more difficult and there is some evidence that the retained metabolic heat is used to raise the local temperature within a bacterial colony when the external temperature decreases [64]. However, quorum sensing in bacteria reduces the metabolic activity proportional to the density of a population [65–67]. It seems plausible that a mechanism akin to bacterial “quorum sensing” must exist in multicellular metazoans to precisely calibrate metabolism and heat production by monitoring the density of a cell population. What would the circuitry of a putative metazoan “quorum sensing” cascade accommodate to achieve this function?

A putative metazoan “quorum sensing” (mQS) pathway would ideally accommodate a linear stoichiometry to precisely convey information regarding the density of a population to the recipient cell. The pathway will be switched on (mQS^on^) via paracrine interactions with a non-diffusible ligand in order to minimise signalling noise. Further, removal of the non-diffusible (i.e. membrane-tethered) ligands after binding to the receptor that activates the mQS cascade enhances the sharpness of generated signals in the putative mQS^on^ state. Finally, the mQS signals must regulate mitochondrial activity and the pathway must be calibrated by negative feedback provided via mitochondrial activity. Notch signalling cascade fulfills a majority of the requirements of a metazoan “quorum sensor” (Fig. 8d).

Notch signalling pathway accommodates a linear hierarchical network topology [4]. The pathway is activated upon association of Notch receptors on recipient cells with membrane tethered ligands on adjacent cells. Upon binding to Notch receptor, endocytosis of the ligand serves the dual purposes of generating the mechanical force to expose the ADAM10 cleavage site and removing the membrane-tethered ligand [10]. Subsequent to activation of the signalling cascade, Notch-1 amplifies mitochondrial activity by canonical [68] and non-canonical [69] upregulation of respiratory chain components. Specifically, expression of cytochrome-c oxidase, the key enzyme in the electron transport chain, is regulated by the Notch1 binding partner RBPJ [70]. By communication with mitochondria, Notch-1 aligns the cellular energetic demand to the anabolic flux, another function that is in part regulated by this signalling pathway [71]. Unlike amplification of mitochondrial activity by Notch signalling, the nature of a potential feedback mechanism that relays information regarding mitochondrial activity to the Notch/RBPJ transcriptional complex to complete the signalling loop, remains unknown. Earlier dissection of the Notch-1 signalling circuitry suggested that individual signalling modules are bistable, and an as-yet-unknown signalling factor is required to complete the signalling circuitry [6]. Here, we provided evidence that thermal energy is consistent with the proposed unknown signal. Subsequent to dismantling of the cell wall integrity pathway, Swi6 was repurposed to a thermodynamic sensor as an integral part of Notch receptors. Enrichment of acidic residues in strategic positions of Notch-1 ankyrin domain facilitates emergence of molecular fluctuations that lead to an emergent entropy of 0.8 (kcal⋅K^−1^.mol^−1^) relative to more stable ankyrin domains of other proteins. Both theoretical and experimental analysis support a model in which a nuclear temperature of 39 °C equivalent to a cytoplasmic temperature of 50 °C is sufficient to destabilize Notch-1 ankyrin domain and terminate the signalling output by dissociation of the transcriptional complex. Structural adaptation to sense stressors is not confined to the ankyrin domain of Notch-1. Modular organisation of ankyrin domain whereby individual modules are connected via flexible β-hairpins gives rise to a molecular nano-spring that converts thermal fluctuations into mechanical energy [33] in neuronal ion channels [34] leading to temperature-dependent channel opening [35]. NF-κB, a key ANK^+^ proinflammatory, senses and responds to thermal fluctuation in a similar manner but in a range close to that of Notch-1 ankyrin [39]. The sensory function of ankyrin domain is not limited to those discussed above. In capillaries, a reduction of oxygen partial pressure is directly sensed by the Band 3–ankyrin complex of RBCs leading to increased deformability of RBCs, increased velocity of blood flow, and re-establishment of the oxygen tension [37]. The sensory activities of ankyrin domain reflect subtle structural modification to utilise the modular organisation of the domain to receive and transmit kinetic energy to adjacent structures. The structural adaptation of Notch-1 ankyrin domain to function as a thermodynamic sensor completes a signalling loop whereby just as Notch-1 amplifies activity of the mitochondrial respiratory chain, resultant thermal energy provides negative feedback to terminate the activity of Notch1 transcriptional complex and to sustain the intracellular temperature within a narrow physiological window. We therefore propose that Notch signalling has evolved to function as a metazoan quorum sensor and the evolutionary adaptations of the ankyrin domain of Notch-1 are central to the activity of this QS pathway.

While the proposed QS mechanism safeguards against random fluctuation of temperature, it also provides a conduit for morphogens to alter mitochondrial activity and Notch signalling output during differentiation. During brain development, cannibalisation of erythroblasts leads to synchronised mitochondrial activation that triggers abrupt inactivation of Notch signalling cascade and accelerated adoption of neuronal fate [15]. This mechanism is compatible with the demonstrated role of mitochondria in neurogenesis [72–75]. Resolution of differentiation/self-renewal dichotomy in cycling neural progenitor cells requires input from mitochondria and a reduction of mitochondrial activity by fusion is a major determinant of sustained self-renewal capacity [76].

## Conclusion and future directions

Findings show that the ankyrin domain of Notch-1 intracellular domain functions as a thermodynamic sensor that inputs heat flux as a proxy for mitochondrial activity in the Notch^on^ state that  is eventually switched off in a temperature-dependent manner. The disclosed mode of communication between mitochondria and the Notch signalling pathway foreshadows the importance of these organelles in regulating the metazoan developmental landscape. While our findings provide evidence for regulation of the canonical Notch signalling pathway via heat flux, it remains unclear whether the ankyrin domain plays a similar role in non-canonical Notch signalling pathways [77]. In non-canonical regulation of β-catenin by Notch-1, the RAM domain plays the key role in stabilising the association of the β-catenin/Notch1 [78]. Could mitochondrial heat flux calibrate the stability of β-catenin/Notch1 assembly? Further experiments are required to answer this question, amongst others, regarding the interface of mitochondrial activity and non-canonical Notch signalling mechanisms.

## Methods

### Reagents

All chemicals were purchased from Sigma-Aldrich Inc. unless stated otherwise. All primers were purchased from IDT DNA.

### Chicken embryos

Fertilised Rhode Island Red eggs were obtained from a local hatchery (Barter & Sons Hatchery). The eggs were transferred to a shell-less culture system after three days and incubated at a temperature of 37 °C and relative humidity of 70%. Experiments were performed on cultivated embryos at defined Hamburger Hamilton stages [79] as per results section. The protocols for this study were approved by the ethics committee of the University of Sydney. All experiments were performed in accordance with guidelines and regulations of University of Sydney and Sydney West Local Health Service.

### Cells

The established mouse multipotent neural stem cells (NSCs: C17.2) were purchased from CellBank Australia (Children’s Medical Research Institute, Australia). Growth medium for NSCs consisted of DMEM/F12 supplemented with 10% fetal Bovine serum and Antibiotic–Antimycotic (100X, Life Technologies). To induce neural differentiation, NSCs were cultured in Knockout™ DMEM (Gibco), GlutaMAX™ supplement, N2 supplement (Gibco), and brain-derived neurotrophic factor (BDNF, 10 ng/ml, Sigma).

### Immunohistochemistry

Specimens were fixed in 2% paraformaldehyde/5% sucrose in 0.02 M phosphate buffer pH: 7.4 (680 mOsm), for 4 h at 4 °C. After blocking in incubation buffer containing 0.1 M PBS, 1% BSA, 0.1% Tween-20, and 5% normal goat serum (for detection with rabbit Abs) or 5% normal rabbit serum (for detection with mouse Abs) for 40 min, sections were incubated with the primary antibodies overnight at 4 ˚C and secondary antibodies for 1 h at room temperature. Specificity controls were carried out by incubating sections with rabbit or mouse IgG negative control antibodies.

### Transmission electron microscopy

For TEM analysis, tissues were fixed in Karnovsky's fixative overnight at room temperature followed by post-fixation in OsO_4_ for 1 h. Preparations were dehydrated in graded alcohols and embedded in low viscosity resin (TAAB Laboratory and Microscopy, United Kingdom). Ultrathin sections were mounted on Pioloform/formvar coated slot grids, stained in uranyl acetate and lead citrate and examined in a Phillips CM120 BioTWIN electron microscope.

### Gene expression analysis

RNA was isolated using RNeasy Mini Kit (Qiagen). After DNase treatment, reverse transcription of the extracted RNA was carried out using a mixture of 1 μL of oligo-dT, 4 μL of total RNA, 1 μL of dNTP Mix (10 mM each), 4 μL of 5 × First-Strand synthesis Buffer, 1 μL of 0.1 M DTT, 1 μL of RNaseOUT (40 units/μL), 1 μL of SuperScript-III reverse transcriptase (200 units). Reverse transcription was performed at 50˚C for 50 min followed by 55˚C for 15 min. RNA was subsequently digested with RNAase H. To design primers, gene sequence data and exon/intron boundaries were obtained from GenBank database (see Table S1). In each of the primer sets, the common 3΄- or 5΄-primer spanned the adjacent exons to prevent amplification of genomic DNA.

### Real-time PCR

Real-time PCR (38 cycles) was performed using SensiFAST™ SYBR® Lo-ROX reagents (BIOLINE®). Reaction mix comprised of 2 μl of cDNA, 400 nM inner primers (1.5 μl/primer), 10 μl of 2 × SensiFAST SYBR Lo-ROX Mix, and 5 μl of PCR-grade water on a Stratagene® Mx3000P real-time PCR instrument. Average efficiency of PCR amplification for each gene of interest was quantified based on a linear regression model using the LineRegPCR software [80]. The relative expression ratio of gene of interest (test:control) was then calculated using the efficiency (Eff.) values based on the method proposed by Pffafi as follows:

$$Ratio = \frac{{(Eff_{{tar}} )^{{\Delta ct_{{tar}} (control-test)}} }}{{(Eff_{{ref}} )^{{\Delta ct_{{ref}} (control-test)}} }}$$

### RNA interference

For small interfering RNA (siRNA)-mediated knockdown of *mm*-notch-1, cells were electroporated with 200 nM of either the targeting or control siRNA. The sequence of siRNA targeting *mm*-notch-1 is as follows:

- sense: 5'-rUrGrCrUrGrCrArCrArCrCrArArCrGrUrGrGrUrCrUrUrCAA-3'.
- antisense: 5'-rUrUrGrArArGrArCrCrArCrGrUrUrGrGrUrGrUrGrCrArGrCrArCrG-3'.

For electroporation in RNAi, cells were harvested, mixed with Dsi-RNA (i.e. siRNA) and resuspended in 400 μL of electroporation buffer (10^6^ cells/400 μL). Electroporation buffer comprised 20 mM HEPES, 135 mM KCl, 2 mM MgCl2, 0.5% Ficoll 400, and 2 mM ATP/5 mM glutathione (pH 7.6). Electroporation was carried out at 1700 V/cm, 700 μs, four pulses at 1-s intervals.

### Molecular beacons

The L-DNA molecular beacons (MB-4 and MB-5) was designed as described elsewhere [52]. Molecular beacons were labelled with a 6-FAM reporter dye at the 5′ end, and an Iowa Black FQ quencher at the 3′ end (Integrated DNA Technologies).

The sequence of MB-4 is as follows:

FAM-CGAGTTTTTTTTTTTTTTTCTCG-IBFQ

The sequence of MB-5 is as follows:

FAM-GCGAGTTTTTTTTTTTTTTTCTCGC-IBFQ

#### Ex ovo electroporation of embryos

ECM 830 electroporator (Harvard Apparatus®) was used to generate square-wave electric pulses. A solution (20 mM HEPES, 135 mM KCl, 2 mM MgCl2, 0.5% Ficoll 400, 2 mM ATP/5 mM glutathione) containing the molecular beacons (200 nM) was injected into the canal of the neural tube under illumination using a surgical microscope (Leica M320). Platinum Tweezertrodes (5 mm, Harvard Apparatus®) were carefully positioned bilaterally around the embryo’s head and 4 mm apart. Parameters for *ex ovo* electroporation of chicken embryos were adapted from Sauka–Spengler et al. [81]. Electroporation was carried out at 62.5 V/cm, 50 ms, 5 pulses at 1-s intervals. The electroporated embryos were then incubated for 24 h at 37 °C before harvesting for histological processing.

#### Adaptive Poisson-Boltzmann solver

The APBS Electrostatics Plugin was accessed from PyMOL (v.2.5). Visualisation of surface potential was also done using PyMOL. All calculations were performed at 0.15 M ionic strength in monovalent salt, 298.15° K, protein dielectric 2, and solvent dielectric 78.

#### Molecular dynamics

The open-source GROMACS tools provided through the Galaxy platform was used to simulate the temperature-dependent dynamics of Notch-1 ankyrin domain (PDB ID: 2F8Y). Simulations were performed using the water model of SPC/E [82] and OPLS/AA force-field [83]. Equilibrating simulations under an NVT (isothermal-isochoric) ensemble, followed by an NPT (isothermal-isobaric) ensemble were performed at 298 K until the root mean-square deviation (RMSD) was stable. The root mean-square fluctuation (RMSF) values were calculated with Gromacs 4.5 through the Galaxy platform. Mutation of ASP-1989 to ASN-1989 in silico (ID: 2F8Y) was performed using PyMOL.

#### Quantification and statistical analysis

SPSS statistical software (SPSS v.16, Chicago, Illinois, US) was used for the statistical analysis of data. The relative expression levels of genes of interest were compared using univariate ANOVA and non-parametric Mann–Whitney U test. The results of statistical analysis can be found in figure legends. Plots were generated using Prism software. Data are presented as Mean ± SD. In the present study, a p-value < 0.01 (*) was considered as statistically significant.

## Supplementary Information

**Additional file 1.** Supplementary information related to the manuscript.

## Data Availability

The published article includes all datasets generated or analyzed during this study.

## Abbreviations

NICD: Notch intracellular domain
ANK: Ankyrin
DSL: Delta-Serrate-Lag
RBP-J: Recombination signal binding protein-J
MAML: Mastermind like transcriptional coactivator
APBS: Adaptive Poisson-Boltzmann solver
