# Supplementary material for: Notch ankyrin domain: evolutionary rise of a thermodynamic sensor
Source: Cell Commun Signal. 2022 May 18;20:66. doi: 10.1186/s12964-022-00886-4 (PMC9118731; doi:10.1186/s12964-022-00886-4)
Supplement: Supplementary file 2 — Additional file 1. Supplementary information related to the manuscript. [file 12964_2022_886_MOESM2_ESM.docx]

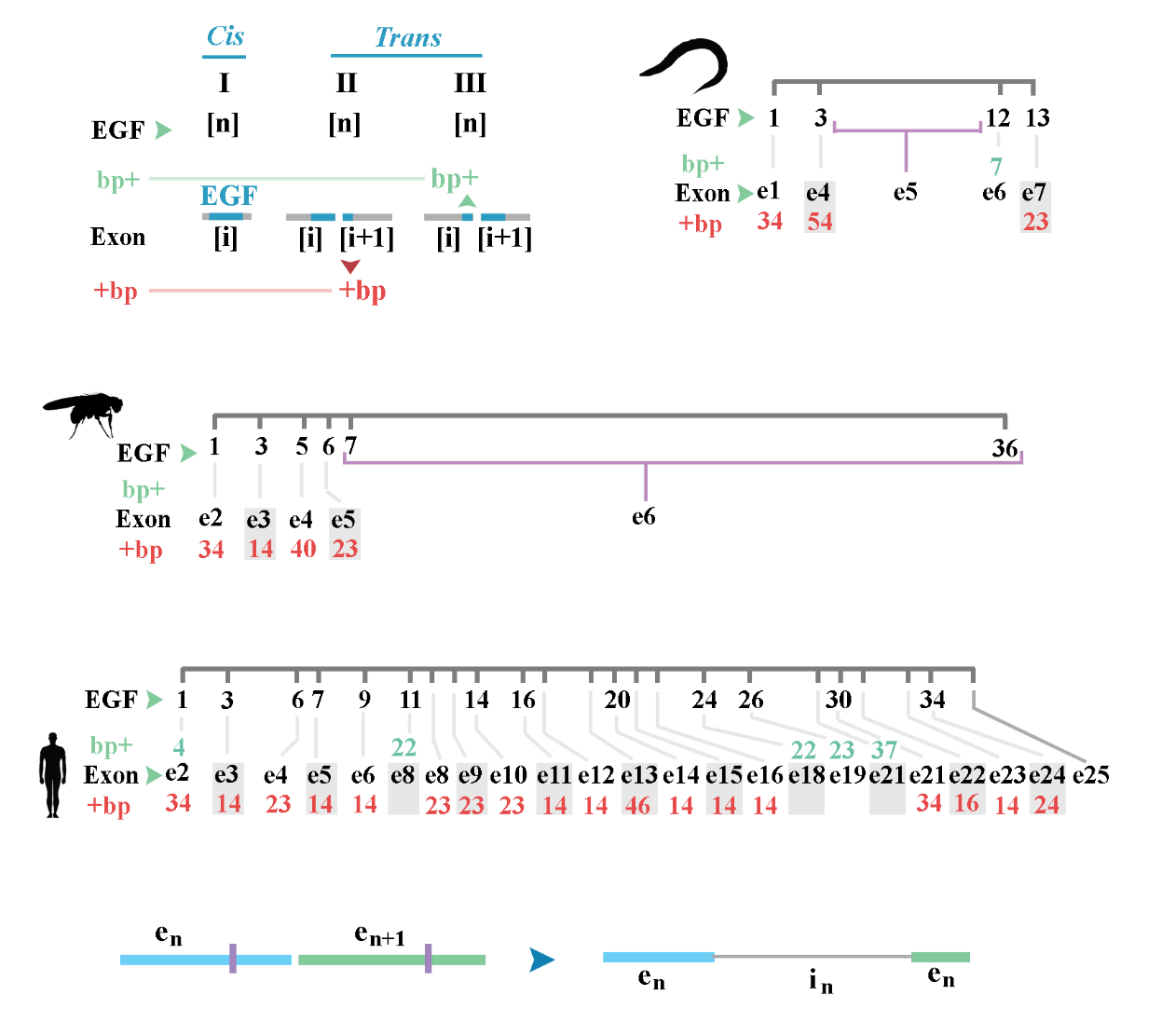


**Figure S1. Genomic evolutionary landscape of Notch-1 EGF domains.** The top left image shows genomic patterns for encoding EGF domains in a single exon (Cis), or as trans-exonic entities. In trans-exonic mode EGF domains are split asymmetrically between two tandem exons. In trans-exonic mode, the exonic fragment encoding the major part of EGF is shown as e[i] and the size of smaller fragment is shown on the top (as green) when it belongs to e[i-1] or at the bottom (red) when it belong to e[i+1]. While Drosophila and C. elegans EGFs are mainly encoded in Cis-mode, most human EGFs are trans-exonic. Further, the patterns of splitting in trans-exonic EGF show similarity between tandem exons. For example, note the similarity of splitting pattern between e8, e9, e10 and also between e14, e15, e16. This observation foreshadows a scenario whereby a combination of genomic duplication and intronization by sacrifice of exons (to generate introns as per bottom diagram) has shaped the genomic landscape of the genomic region encoding EGF domains in vertebrates. Radical divergence of evolution provides a potential explanation for low homology of amino acid sequences that belong to human and drosophila EGF domains.


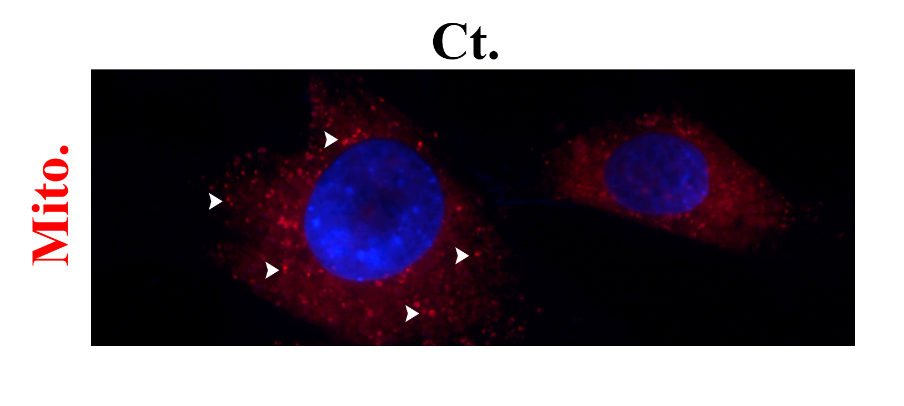


**Figure S2.** Application of MitoTracker red show few active mitochondria scattered throughout the cytoplasm in control Heme^-^ cells.


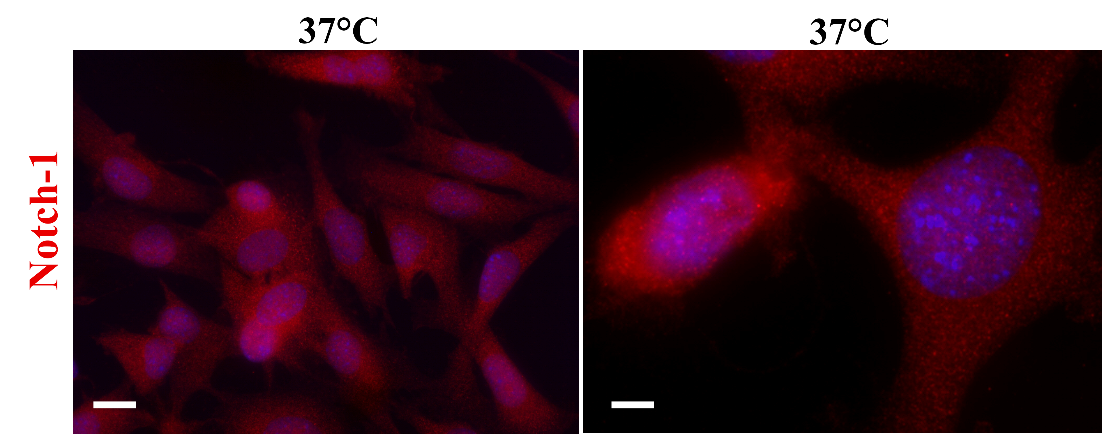


**Figure S3.** Immunohistochemical visualisation of intracellular Notch-1 in neural progenitor cells at 37°C. Scale bars: left 20µm, right 5µm.
